# Comparison of the practicability of ultrasound and spiral computed tomography in the diagnosis of colon cancer: A protocol for systematic review and meta-analysis

**DOI:** 10.1097/MD.0000000000023381

**Published:** 2020-12-11

**Authors:** Fan Zhao, Qingya Yang, Cunzhong Meng, Tao Jiang

**Affiliations:** Wuwei People's Hospital, Wuwei, Gansu Province, China.

**Keywords:** colon cancer, protocol, spiral computed tomography, systematic review, ultrasound

## Abstract

**Background:**

Colon cancer is a common malignant tumor of the gastrointestinal tract. Therefore, a clear diagnosis is particularly important for the treatment of colon cancer. Ultrasound and spiral computed tomography (CT) can both be used in the diagnosis, but each has its own advantages and disadvantages, which could cause confusion in clinical choice. The purpose of this study was to systematically evaluate the practicability of spiral CT and ultrasound in the diagnosis of colon cancer.

**Methods:**

A systematic search was performed by retrieving on English databases (PubMed, Embase, Web of Science, the Cochrane Library) and Chinese databases (CNKI, Wanfang, Weipu [VIP], CBM). Besides, manually search for Google and Baidu academic of diagnostic experimental study of ultrasound and spiral CT in the diagnosis of Colon Cancer. The retrieval time limit was from the establishment of the database to October 2020. Two researchers independently extracted and evaluated the quality of the data in the included study. A meta-analysis was performed using Meta Disc1.4 and RevMan5.3 software.

**Results:**

Sensitivity, specificity, positive Likelihood ratio, negative likelihood ratio, and diagnostic odds ratio were used to determine the diagnostic efficacy of ultrasonography and helical CT in colorectal cancer.

**Conclusions:**

This study will compare the practicability of CT and ultrasound in the diagnosis of colon cancer and provide reliable evidence-based basis for clinicians to choose the appropriate or best evidence-based basis.

**Ethics and dissemination:**

The private information from individuals will not be published. This systematic review also will not involve endangering participant rights. Ethical approval is not required. The results may be published in a peer-reviewed journal or disseminated in relevant conferences.

**OSF Registration number:**

DOI 10.17605/OSF.IO/WAJHQ

## 1 Introduction

Colon cancer is a common malignant tumor of digestive system with high morbidity and mortality. In the United States, colorectal cancer is the fourth most commonly diagnosed cancer and the second leading cause of cancer death.^[1]^ In China, the incidence and mortality of colorectal cancer rank third and fifth among all malignant tumors.^[2]^ As the early symptoms are not obvious, hematoma, diarrhea, change of bowel habits, local abdominal pain, anemia, and other symptoms occur along with the increase of cancer, and most of the patients’ early symptoms are not easily detected. In the past 20 years, about 20% of the patients have reached the late stage of clinical diagnosis and lost the best time for treatment.^[3]^ The 5-year survival rate of colon cancer after radical resection is 60% to 80%, so early detection and treatment are of great significance for improving prognosis.^[4]^ Colonoscopy and barium meal examination are commonly used in the diagnosis of colon cancer.^[5]^ However, it is difficult to determine the depth and range of invasion, which may cause discomfort to patients in the meanwhile. Clinical studies have shown that the depth of invasion of colon cancer, the presence or absence of lymph node metastasis and distant metastasis are the key factors affecting the clinical prognosis of rectal cancer.^[6]^ Therefore, the application of imaging methods to fully demonstrate the anatomical relationship of the pelvic cavity, accurately evaluate and summarize the tumor stage, surrounding anatomical relationship and metastasis is of great value for the reasonable formulation of treatment and improvement of clinical prognosis. CT is currently the main method for the diagnosis and staging of colon cancer. However, due to the limited contrast of soft tissue, its staging of primary tumor (Stage T) or detection of extramural invasion are generally unsatisfactory, with an accuracy rate between 60% and 80%.^[7–9]^ Meanwhile, it has a low accuracy in detecting nodal involvement,^[10]^ and is expensive and cannot be widely used in the early stages of the disease. Ultrasound is widely used in the general survey and screening of parenchymal organs due to its advantages of low price, non-invasiveness, simplicity, and strong repeatability.^[11]^ Due to the interference of gas, ultrasound has long been considered as a difficult examination method to determine the details of the lesions. With the progress of technology and the improvement of image quality, ultrasound is becoming both convenient and affordable, and the diagnosis of colon cancer by ultrasound technology has once again attracted the attention of clinicians.

Although a number of experimental studies have compared the practicality of ultrasound and CT in the diagnosis of colon cancer,^[12–14]^ the conclusions are not consistent. The purpose of this systematic evaluation is to evaluate the reliability of 2 imaging methods in the diagnosis of colon cancer and to provide an evidence-based basis for clinicians.

## 2 Methods

### 2.1 Protocol register

This protocol of systematic review and meta-analysis has been drafted under the guidance of the preferred reporting items for systematic reviews and meta-analyses protocols (PRISMA-P).^[15]^ And, it has been registered on the open science framework (OSF) on October 24, 2020. (Registration number: DOI 10.17605/OSF.IO / WAJHQ).

### 2.2 Ethics

Since this is a protocol with no patient recruitment and personal information collection, the approval of the ethics committee is not required.

### 2.3 Eligibility criteria

#### 2.3.1 Types of studies

We will collect case–control studies and cohort studies of ultrasound compared with spiral CT in the diagnosis of colon cancer. Regardless of blinding, publication status, region, but Language will be restricted to Chinese and English.

#### 2.3.2 Objects of studies

Patients were diagnosed with colon cancer by ultrasound or CT and confirmed by pathological examination as the gold standard. There were no restrictions on nationality, race, age, sex, course of disease, etc.

#### 2.3.3 Types of tests

The observation group and control group were examined by ultrasound and CT respectively, and there was no limitation on the type of examination equipment. All patients underwent pathological examination to evaluate the accuracy of the imaging examination.

#### 2.3.4 Types of outcome indicators

Sensitivity (SEN), specificity (SPE), positive likelihood ratio (+LR), negative likelihood ratio (–LR), diagnostic odds ratio (DOR), summery receiver operating characteristic curve (SROC), area under curve (AUC) of ultrasound, or spiral CT in the diagnosis of colon cancer.

### 2.4 Exclusion criteria

Studies published repeatedly; studies whose literature are abstract or data are incomplete, or whose data could not be obtained after contacting the author; abstracts, comments, reviews, case reports, etc; studies with obvious data errors; studies without gold standard verification.

### 2.5 Search Strategy

“Ultrasound” (chao sheng), “Spiral CT” (luo xuan CT), “Colon neoplasms” (jie chang zhong liu), “Colon cancer” (jie chang ai), “Colorectal cancer” (da chang ai) were used for retrieval in Chinese databases, including CNKI, Wanfang Data Knowledge Service Platform, VIP Information Chinese Journal Service Platform, and China Biomedical Database. English retrieval words such as “Ultrasound,” “Spiral CT,” “Spiral Computed Tomography,” “Colonic Neoplasm,” “Colon Cancer” were used for retrieval in English databases, including PubMed, EMBASE, Web of Science, and the Cochrane Library. The retrieval time was from the establishment of the database to October 2020, and all the domestic and foreign literatures about the diagnosis of colorectal cancer by ultrasound contrast spiral CT were collected. Take PubMed as an example, and the retrieval strategy is shown in Table 1.

**Table 1:** Search strategy in PubMed database.

Number	Search terms
#1	Ultrasound [Title/Abstract]
#2	Spiral CT [Title/Abstract]
#3	Spiral Computed Tomography [Title/Abstract]
#4	#2 OR #3 OR
#5	Colon Cancer [MeSH]
#6	Colonic Neoplasm [Title/Abstract]
#7	Neoplasm, Colonic [Title/Abstract]
#8	Colon Neoplasm [Title/Abstract]
#9	Neoplasm, Colon [Title/Abstract]
#10	Cancer of Colon [Title/Abstract]
#11	Colonic Cancer [Title/Abstract]
#12	Cancer, Colonic [Title/Abstract]
#13	#5 OR #6 OR #7 OR #8 OR #9 OR #10 OR #11 OR #12
#14	#1 AND #4 AND #13

### 2.6 Data screening and extraction

**Figure 1:**
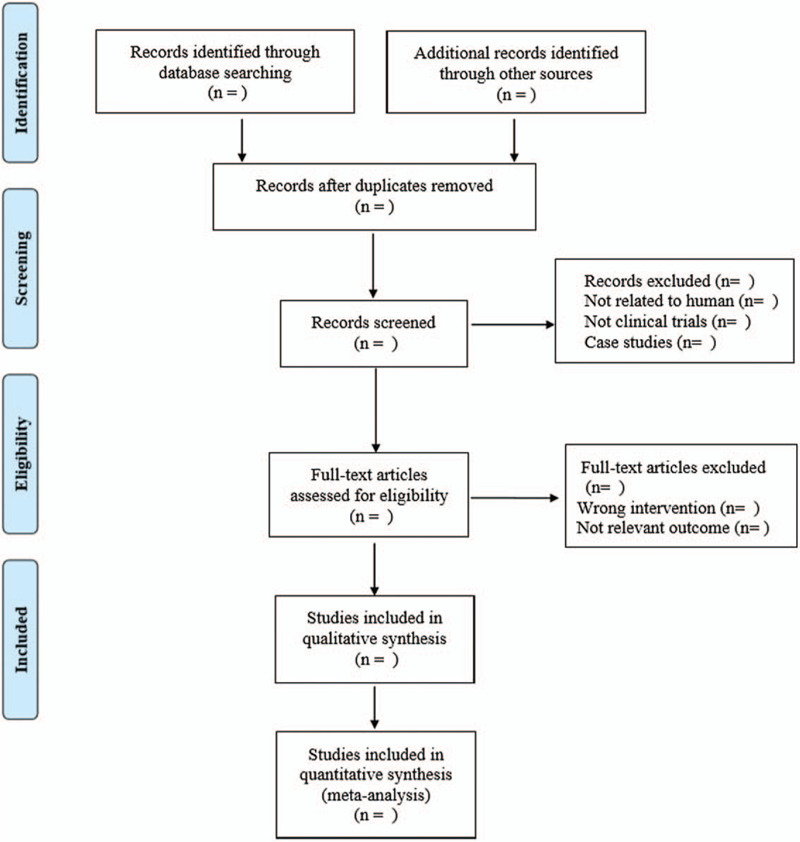
Flow diagram.

Referring to the method of research selection in version 5.0 of the Cochrane collaboration Network system Evaluator Manual, according to the preferred reporting items for systematic reviews and meta-analyses (PRISMA) flow chart, the 2 researchers used the EndNote X9 document management software to independently screen and check the literature according to the above inclusion and exclusion criteria, and check each other, if there were different opinions, negotiate with a third party to resolve the differences. According to the inclusion and exclusion criteria of the literature, 2 researchers independently extracted relevant data from each eligible study and recorded them through Excel 2013. Basic features of the included studies, including first author, year of publication, language, research country, number of experimental cases, imaging method, gold standard, etc. Key elements of bias risk assessment. The outcome measurement index data concerned, such as true positive value, false positive value, true negative value, false negative value, etc. The literature screening process is shown in Fig. 1.

### 2.7 Literature quality assessment

QUADAS-2 quality evaluation criteria^[16]^ were used to evaluate the risk of bias in the included literature. The tool consists of 2 aspects: bias evaluation and applicability, including case selection, trial to be evaluated, gold standard, case flow, and progress. The choices of “high risk,” “low risk,” and “unclear” are given respectively for the items covered by all areas. Risk bias evaluation was given by the 2 researchers according to the performance of the included literature in the above evaluation items, and cross-checked after completion respectively. In case of any disagreement, discussion was required. If no agreement could be reached, a decision would be made in consultation with researchers from the third party.

### 2.8 Statistical analysis

#### 2.8.1 Data analysis and processing

Meta analysis was performed using RevMan 5.3 software and Meta Disc 1.4 software. Heterogeneity was determined by *I*^2^ values. If (*P* ≥ .1, *I*^*2*^ ≤ 50%), there was low inter-study heterogeneity, and the fixed-effect model was adopted to conduct a meta-analysis. If (*P *< .1, *I*^2^ > 50%), it indicated inter-study heterogeneity and should explore the source of heterogeneity. Calculate the sensitivity (SEN), specificity (SPE), positive likelihood ratio (+LR), negative likelihood ratio (–LR), diagnostic odds ratio (DOR), and its 95% confidence interval (CI), draw summary receiver operating characteristic curve (SROC), and obtain area under curve (AUC). At the same time, the sensitivity analysis of the literature was excluded one by one to evaluate the stability of the research results.

#### 2.8.2 Dealing with missing data

If there is missing data in the article, contact the author via email for additional information. If the author cannot be contacted, or the author has lost relevant data, descriptive analysis will be conducted instead of meta-analysis.

#### 2.8.3 Subgroup analysis

This study will carry out a subgroup analysis based on the different patient characteristics, index and reference tests, and outcome indicators.

#### 2.8.4 Assessment of publication bias

If there are >10 studies, the Deek funnel plot will be used to assess potential publication bias. Moreover, Egger and Begg test were used for the evaluation of potential publication bias.

#### 2.8.5 Grading the quality of evidence

We will use Grading of Recommendation Assessment, Development and Evaluation (GRADE) scoring method to grade the evidence of the outcome index.^[18]^ The evaluation includes bias risk, indirectness, inconsistency, inaccuracy, and publication bias. The quality of evidence will be rated as high, medium, low, or very low.

## 3 Discussion

Due to the lack of typical clinical symptoms and signs in the early stage of colon cancer, early diagnosis and differentiation is relatively difficult. CT is a common method for the diagnosis and staging of colon cancer. It has advantages in detecting extramural invasion and lymph node metastasis of colon cancer^[19]^ and is highly sensitive to tumor invasion of colon cancer beyond the intestinal wall (T1–T2 vs T3–T4). However, it remains a challenge to detect tumor invasion of 5 mm or more (T1–T3ab vs T3cd–T4).^[20]^ There are also problems of radiation damage and high cost.

The advantages of ultrasound diagnosis of colon cancer are as follows: as a non-invasive examination method, ultrasound is relatively inexpensive and is not limited by time and place. It is easy to be accepted by patients and can be used as the preferred screening method for colon cancer.^[21]^ For patients with clinical findings of abdominal mass, the location, shape, size, internal echo, boundary, mobility, and the relationship with surrounding organs of the mass can be clearly displayed, as well as whether there is invasion and metastasis.^[22]^

Both ultrasound and spiral CT have their own advantages in the diagnosis of colon cancer. How to choose an appropriate examination scheme to maximize the benefit of both patients and decision-making doctors is an urgent problem to be solved. This systematic review hopes to provide effective information for clinicians to understand the practicability of the 2 imaging methods, and to provide the best way for the diagnosis of colon cancer patients.

However, this systematic review has some limitations. Different types of equipment, frequency of ultrasound probe, and thickness of spiral CT scan used in the included studies may cause some clinical heterogeneity. In addition, due to the limitation of language ability, we only search English and Chinese literature and may ignore studies or reports in other languages.

## Author contributions

**Data curation:** Fan Zhao, Qingya Yang.

**Funding acquisition:** Qingya Yang.

**Literature retrieval:** Fan Zhao and Qingya Yang.

**Resources:** Fan Zhao, Qingya Yang.

**Software:** Cunzhong Meng.

**Supervision:** Tao Jiang.

**Writing – original draft:** Fan Zhao, Tao Jiang.

**Writing – review & editing:** Fan Zhao, Qingya Yang.
